# Quantum-inspired adaptive simulated annealing for antenna selection and joint optimization in RIS-assisted MIMO-NOMA systems

**DOI:** 10.1038/s41598-026-47710-4

**Published:** 2026-04-28

**Authors:** Samar I. Farghaly, Heba S. Dawood, Hager S. Fouda

**Affiliations:** Department of Electronics and Electrical Communications Engineering, Faculty of Engineering, Tanta University, Tanta, 31111 Egypt

**Keywords:** Energy Efficiency (EE), Joint Optimization, Multiple-input Multiple-output (MIMO), Reconfigurable Intelligent Surfaces (RIS), Simulated Annealing (SA), Spectral Efficiency (SE), Engineering, Mathematics and computing, Physics

## Abstract

Multiple-input multiple-output non-orthogonal multiple access (MIMO-NOMA) systems assisted by reconfigurable intelligent surfaces (RIS) have become a viable way to improve next-generation wireless networks. However, MIMO beamforming, antenna selection (AS), RIS phase configuration, and NOMA power allocation are governed by intricate and closely related relationships. Consequently, attaining optimal performance within these integrated systems remains very difficult. Especially in large-scale MIMO deployments, the resulting joint optimization problem is computationally demanding, multidimensional, and non-convex. Conventional convex optimization techniques and heuristic algorithms frequently struggle with slow convergence, local optima, and high computational burden. However, the practical applicability of these methods is significantly limited. This paper presents AQSA algorithm that simultaneously optimizes MIMO beamforming, NOMA power allocation, RIS phase shifts, and AS. It is performed in order to overcome these difficulties. An adaptive temperature control mechanism dynamically balances exploration and exploitation during the optimization process. In addition, the proposed AQSA framework integrates quantum computing concepts like superposition and probabilistic state transitions to improve search capability. Better convergence speed, resilience, and adaptability to various network configurations are guaranteed by this hybrid design. The performance of the suggested method is assessed using extensive numerical simulations in a range of system conditions. The findings show that, when compared to traditional approaches, the AQSA-based joint optimization considerably increases system sum-rate, energy efficiency (EE), and user fairness, while keeping the computational complexity manageable. Additionally, the framework can efficiently utilize the extra degrees of freedom offered by larger antenna arrays and RIS deployments. As well, it is scalable to large-scale MIMO–NOMA systems. These results demonstrate how the proposed work achieves noticeable enhancement in spectral effeciency (SE).

## Introduction

The rapid advancement of next-generation wireless communication systems requires the development of innovative technologies capable of achieving high spectral efficiency, massive connectivity, and energy-efficient transmission^1–4^. MIMO systems have long been recognized as a key technology for meeting these requirements by exploiting spatial multiplexing and diversity gains^5–7^. However, the performance of conventional MIMO systems is often constrained by high hardware complexity, increased power consumption, and unfavorable propagation environments. To address these challenges, Non-Orthogonal Multiple Access (NOMA) and Reconfigurable Intelligent Surfaces (RIS) have recently emerged as promising enabling technologies for beyond-5G and 6G wireless networks^8–12^. RIS technology employs a nearly passive and reconfigurable metasurface capable of intelligently controlling incident electromagnetic waves through programmable phase shifts^13–15^. By appropriately adjusting the reflection coefficients of its elements, RIS can effectively reshape the wireless propagation environment, enhance signal strength, and mitigate interference. In parallel, NOMA improves spectral efficiency and user connectivity by allowing multiple users to share the same time–frequency resources through power-domain multiplexing. Therefore, integrating MIMO, RIS, and NOMA forms a powerful multi-layer communication architecture that jointly enhances channel conditions, improves user multiplexing, and enables more efficient resource allocation^16,17^.

### Related work

#### antenna selection in large-scale MIMO systems

Antenna selection (AS) has been widely investigated as an efficient technique for reducing hardware complexity and power consumption in large-scale MIMO systems while preserving most of the achievable diversity and multiplexing gains. In^18^, a full-duplex cooperative NOMA system was studied in which a multi-antenna relay connects a multi-antenna base station to remote users. Two AS schemes, namely max-U1 and max-U2, were proposed to maximize the end-to-end SINR for near and far users, respectively. In addition, a two-stage QoS provisioning strategy was introduced to guarantee the minimum rate requirement of the far user while maximizing the near user’s throughput. To further enhance system performance, dynamic antenna clustering was employed to adaptively divide relay antennas into transmit and receive subsets. Closed-form expressions for outage probability and achievable rate were derived to evaluate the proposed schemes. Although these techniques effectively improve system performance, they primarily focus on relay-assisted architectures and do not consider programmable wireless environments enabled by emerging RIS technology.

#### Beam and antenna selection for massive MIMO-NOMA systems

Beam and antenna selection techniques have also been investigated to support NOMA transmission in massive MIMO systems. In^19^, a beam selection strategy based on intra-beam superposition coding was proposed for downlink massive MIMO systems employing analog beamforming and NOMA. The proposed approach aims to select beams with a large number of candidate users while minimizing interference among simultaneously active beams. To address the limited availability of channel state information caused by long feedback intervals, the authors introduced an angle-domain grouping (ADG) method that considers both candidate beams and neighboring beams during the selection process. In addition, SINR-based scheduling was incorporated to improve transmission rate control. Despite the effectiveness of these beam-domain optimization techniques, they mainly rely on conventional beamforming strategies and do not exploit the potential of reconfigurable propagation environments.

#### RIS-assisted MIMO systems with antenna selection

Recently, reconfigurable intelligent surfaces (RIS) have attracted significant attention as a promising technology for enhancing wireless communication performance by dynamically controlling electromagnetic wave propagation. In^20^, the authors proposed a RIS-assisted massive MIMO architecture incorporating antenna selection to mitigate the performance degradation caused by reducing the number of active antennas. By adjusting the reflection coefficients of RIS elements, the wireless propagation environment can be intelligently modified to compensate for the loss in array gain introduced by antenna selection. Simulation results demonstrated that RIS can significantly enhance spectral efficiency while maintaining low hardware complexity. However, jointly optimizing antenna selection and RIS configuration remains a challenging problem due to the large search space and high computational complexity involved.

#### Optimization techniques for RIS–NOMA systems

Several studies have investigated optimization strategies for RIS-assisted NOMA systems. In^21^, the authors analyzed downlink communications in sparsely deployed RIS-assisted NOMA systems and proposed a multi-stage optimization framework. The framework integrates simulated annealing for RIS deployment, integer programming for power allocation, inequality-based reflection coefficient design, and genetic algorithms for channel assignment. Simulation results showed that the proposed scheme achieves up to 30% throughput improvement compared with conventional RIS-assisted orthogonal multiple access systems. Nevertheless, the complexity of such multi-stage optimization methods may limit their scalability in practical large-scale systems. Other studies have explored the integration of RIS with antenna selection or beamforming strategies to further improve system performance. For instance,^22^ proposed RIS-assisted receive spatial modulation systems combined with several antenna selection methods, including capacity-optimized antenna selection (COAS), antenna correlation-based antenna selection (ACAS), and Euclidean distance-based antenna selection (EDAS). These approaches demonstrated improved error performance compared with conventional RIS-assisted systems. Additionally, the work in^23^ investigated the joint optimization of beamforming and antenna selection for spatially distributed array radar systems, while^24^ studied antenna-related optimization for high-precision indoor localization systems. Despite these advancements, most existing approaches rely on classical optimization algorithms that may suffer from high computational complexity and slow convergence when applied to large-scale RIS-assisted networks.

#### Research gap and motivation

From the above discussion, it can be observed that existing studies have explored antenna selection, beam selection, and RIS-assisted communication techniques separately or through conventional optimization frameworks. However, the joint optimization of antenna selection and RIS configuration in RIS–NOMA systems remains a challenging problem due to the high-dimensional search space and the strong coupling between system parameters. Furthermore, traditional optimization methods often exhibit high computational complexity and limited scalability in large-scale networks. To address these challenges, this paper proposes an Adaptive Quantum inspired Simulated Annealing (AQSA)-based optimization framework for RIS-assisted MIMO–NOMA systems. The proposed method aims to efficiently optimize antenna selection and RIS configuration simultaneously while maintaining manageable computational complexity and improving overall system performance. In this paper, we provide a low-complexity antenna selection-based positioning technique to balance computational complexity or hardware cost with positioning performance. With the aid of the theoretical error-bound analysis, the ideal antenna subset is chosen, guaranteeing stable and accurate positioning performance. The numerical findings demonstrate that the suggested AS process can save the improvement in positioning accuracy brought about by the spatial domain observations while reducing the implementation complexity in terms of running times by more than 100–200 times. Furthermore, the authors in ^25^ present the AS technique in satalite where for communication between uncrewed aerial vehicles (UAVs) and low Earth orbit (LEO) satellites, we suggest a transmit antenna selection (TAS) technique based on gated recurrent units (GRUs). By collecting the multiscale, time-varying channel quality of the downlink (i.e., from the LEO satellite to the UAV), the GRU network is able to forecast the post-processed SNR for each antenna with high accuracy. The UAV can choose the right antenna indices for upcoming uplink transmissions (i.e., from the UAV to the LEO satellite) based on the projected pSNR. As a result, the pSNR may be raised, which will enhance the UAV and LEO satellite’s communication capabilities.

### Contributions

Nevertheless, it is very challenging to get optimal performance in such systems because to the complex connection between MIMO beamforming, antenna selection, RIS phase setup, and NOMA power allocation. It is a computationally intensive and non-convex joint optimization issue, especially in large-scale MIMO installations. Achieving a balance between system complexity and performance also requires selecting the appropriate antennas, which may significantly reduce hardware costs and power consumption while maintaining virtually optimal transmission quality. This multidimensional optimization is frequently difficult for conventional convex optimization techniques and heuristic search algorithms to manage effectively, leading to slow convergence and local minima. In order to overcome these obstacles, this paper presents the AQSA algorithm^26–28^, which simultaneously optimizes NOMA power allocation, RIS phase shifts, antenna selection, and MIMO beamforming. The suggested AQSA^29^ framework uses quantum computing concepts like superposition and probabilistic state transitions to improve global search capabilities. During the optimization process, an adaptive temperature control mechanism dynamically strikes a balance between exploration and exploitation. Better convergence speed, resilience, and adaptability to various network configurations are guaranteed by this hybrid design. The main contributions of this work are summarized as follows:

- We propose a novel framework for simultaneous antenna selection, RIS phase-shift optimization, and NOMA power allocation in MIMO–NOMA systems, addressing a gap in the existing literature. This framework is supported by clear modeling of the effective channel and system constraints.
- The AQSA algorithm is introduced to efficiently solve the joint optimization problem. Its role in each optimization step is explicitly described, ensuring transparency and reproducibility.
- The proposed approach significantly improves system sum-rate, energy efficiency, and user fairness, demonstrating the benefits of integrating RIS with MIMO–NOMA systems under realistic propagation conditions.
- Despite the complexity of the joint optimization problem, the AQSA-based method maintains manageable computational complexity, making it suitable for practical large-scale implementations.
- Comprehensive numerical simulations validate the effectiveness, robustness, and scalability of the proposed method across various system scenarios, with each result clearly linked to the system parameters and assumptions.
- The proposed algorithm is applicable for Internet of Things (IoT) applications and future 6G communications, highlighting its practical relevance.

## System model

**Figure 1:**
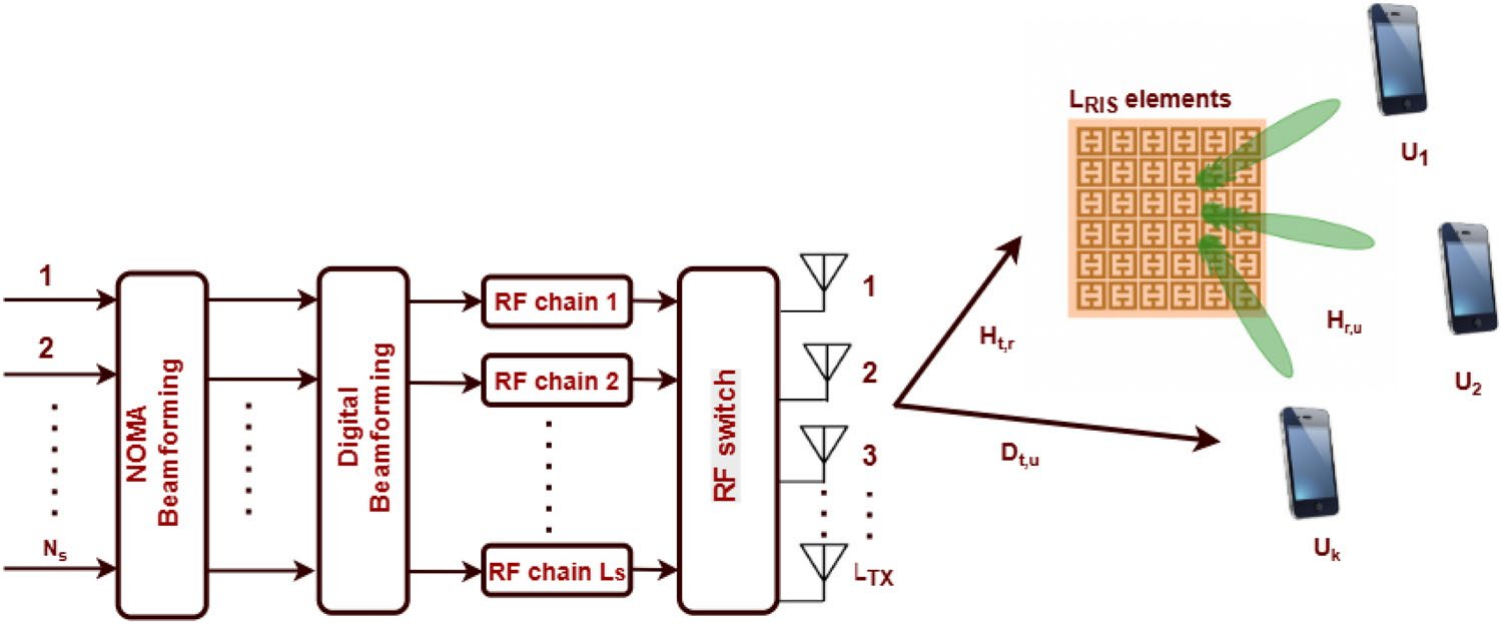
System model of MIMO with RIS-aided system.

Consider a down link NOMA-MIMO wireless communication system assisted by a RIS as shown in Fig. 1. This system is applied to a multi-user system. The proposed system consists of BS with $L_{TX}$ antennas communicating with *K* single-antenna users. The link between the BS and the user *k* is assisted by RIS, which has $L_{RIS}$ reflecting elements. The strategic deployment of the RIS aims to improve communication quality by intelligently manipulating the wireless propagation. The communication region has many items that are environmentally obstructive, which may hinder the LoS pathways between users and the BS. The received signal at the *k*-th user is given by:

1 $$\begin{aligned} \textbf{y}_k = \textbf{d}_{t,u}^H \textbf{x} + \textbf{h}_{r,u}^H \boldsymbol{\Theta } \textbf{H}_{t,r}\textbf{x} + n_k \end{aligned}$$

where $\textbf{d}_{t,u} \in \mathbb {C}^{L_TX \times 1}$ is the direct channel between BS and user *k*, $\textbf{H}_{t,r} \in \mathbb {C}^{L_{\text {RIS}} \times L_TX}$ is the BS–RIS channel, $\textbf{h}_{r,u} \in \mathbb {C}^{L_{\text {RIS}} \times 1}$ is the RIS–user channel, $\boldsymbol{\Theta } = \text {diag}(e^{j\theta _1}, e^{j\theta _2}, \ldots , e^{j\theta _{L_{\text {RIS}}}})$ is the RIS phase shift matrix, and $n_k \sim \mathcal{C}\mathcal{N}(0, \sigma ^2)$ is the additive white Gaussian noise. Furthermore, the transmitted signal *x* from the BS antennas is the superposition of all precoded user signals as the following:

2 $$\begin{aligned} \textbf{x} = \sum _{k=1}^K \textbf{w}_k s_k = \textbf{W} \textbf{s} \end{aligned}$$

where *W* is denoted by $\textbf{W} = [\textbf{w}_1, \ldots , \textbf{w}_K] \in \mathbb {C}^{L_{Tx} \times K}$, where $\textbf{w}_k \in \mathbb {C}^{L_{Tx} \times 1}$ is the precoding vector for user *k*. Additionally $\textbf{s} = [s_1, \ldots , s_K]^T$ is express the vector of *K* independent transmitted symbols, where $s_k$ is the symbol for the user *k* is following ($\mathbb {E}[|s_k|^2] = 1$). Finally, the total transmit power constraint is $P_{\text {Tx}} = \mathbb {E}[\Vert \textbf{x}\Vert ^2] = \text {Tr}(\textbf{W}\textbf{W}^H) \le P_{\max }$. The channel model is presented in the following subsection.

The next subsection will describe antenna selection strategy.

### Antenna selection technique

In huge MIMO systems, turning on all transmit antennas simultaneously increases the hardware complexity, energy consumption, and channel estimation overhead. Antenna selection (AS) algorithms are used to choose a subset of transmit antennas that provide the optimal balance between energy efficiency and spectrum efficiency. Despite the base station having $L_{TX}$ transmit antennas, only $L_s$ $(L_s < L_{TX})$ are engaged at each transmission. Give an example of a binary selection vector.

3 $$\begin{aligned} \textbf{a} = [a_1, \quad a_2, \dots , \quad a_{L_{s}}] \end{aligned}$$

where

4 $$\begin{aligned} a_i = {\left\{ \begin{array}{ll} 1, & \text {if the { i}-th antenna is selected}, \\ 0, & \text {otherwise.} \end{array}\right. } \end{aligned}$$

The effective channel matrix after selection can be expressed as

5 $$\begin{aligned} \textbf{H}_{\text {eff}} = \textbf{H} \textbf{AS}, \end{aligned}$$

Following Eq. (5), the following clarification is provided. Here, $\textbf{AS} \in \mathbb {R}^{L_S \times L_S}$ denotes the antenna selection matrix, defined as $\textbf{AS} = \textrm{diag}(\textbf{a})$, where $\textbf{a} = [a_1, a_2, \ldots , a_{L_S}]$ is the binary antenna selection vector. Each element $a_i \in \{0,1\}$ indicates whether the *i*-th antenna is selected. Note that $\textbf{AS}$ is a single diagonal matrix and does not represent the product of two separate matrices. In addition, $\textbf{H}$ is defined as the following:

6 $$\begin{aligned} \textbf{H} \;=\; \textbf{D}_{\textrm{t,u}} \;+\; \textbf{H}_{\textrm{t,r}} \, \boldsymbol{\Theta } \, \mathbf {H_{\textrm{r,u}}}. \end{aligned}$$

where $\textbf{H}$ is the composite channel matrix incorporates both the direct and IRS-assisted links where $\textbf{D}_{t,u}$ is constructed from the direct channel vectors $\boldsymbol{d}_{t,u}$. As well, $\textbf{H}_{t,r}$ and $\textbf{H}_{r,u}$ denote the BS-IRS and IRS-UE channel matrices, respectively. The direct channel matrix is defined as:

7 $$\begin{aligned} \textbf{D}_{t,u} = \big [ \boldsymbol{d}_{t,u}^{(1)}, \boldsymbol{d}_{t,u}^{(2)}, \dots , \boldsymbol{d}_{t,u}^{(K)} \big ] \in \mathbb {C}^{L_{Tx} \times K} \end{aligned}$$

### Channel model

The channel is modeled as a direct channel model and cascaded channel as the following:

#### Direct channel model

The direct channels between *K* users and BS can be denoted as $\boldsymbol{D} = [\boldsymbol{d}_1, \boldsymbol{d}_2, \ldots , \boldsymbol{d}_K] \in \mathbb {C}^{L_{Tx} \times K}$. These channels can be modeled as a Rayleigh fading model ^20,22^. The channel for the user $k$ is denoted by $\boldsymbol{d}_k^T = [\boldsymbol{d}_{1,k}^T, \boldsymbol{d}_{2,k}^T, \ldots , \boldsymbol{d}_{L_{TX},k}^T] \in \mathbb {C}^{1 \times L_{TX}}$ where the direct channel can be modeled as:

8 $$\begin{aligned} \boldsymbol{d}_{t,u} = \sqrt{\beta _{t,u}} \tilde{\boldsymbol{d}}_{t,u} \in \mathbb {C}^{L_{Tx} \times 1} \end{aligned}$$

where $\tilde{\boldsymbol{d}}_{t,u}$ represents the NLoS component of the direct channel between the BS with $L_{TX}$ transmit antennas and user $k$, and $\beta _{t,u}$ represents the large-scale path loss. The components of $\tilde{\boldsymbol{d}}_{u,t}$ are the corresponding small-scale fading vector with normalized power, and they are complex Gaussian random variables that are independent and identically distributed (i.i.d.), which $\tilde{\boldsymbol{d}}_{t,k} \sim \mathcal{C}\mathcal{N} (\boldsymbol{0}, \boldsymbol{I}_{L_{TX}})$.

#### cascaded channel model(RIS-assisted channel)

The BS-to-RIS channel is denoted as $\textbf{H}_{t,u}$ while the channel between RIS and the user is represented by $\textbf{H}_{r,u}$. These channels are modeled as Rician fading channels and can be written as the following:

9 $$\begin{aligned} \textbf{H}_{t,r}&= \sqrt{\frac{K_g}{K_g+1}} \textbf{H}_{t,r}^{\text {LOS}} + \sqrt{\frac{1}{K_g+1}} \textbf{H}_{t,r}^{\text {NLOS}},\end{aligned}$$

10 $$\begin{aligned} \textbf{H}_{r,u}&= \sqrt{\frac{K_r}{K_r+1}} \textbf{H}_{r,u}^{\text {LOS}} + \sqrt{\frac{1}{K_r+1}} \textbf{H}_{r,u}^{\text {NLOS}}, \end{aligned}$$

where $K_g$ and $K_r$ are the Rician factors, and $\textbf{H}_{t,r}^{\text {LOS}}$ and $\textbf{H}_{r,u}^{\text {LOS}}$ are the LOS components. Consequently, they can be constructed using array response vectors as follows:

11 $$\begin{aligned} \textbf{a}(\phi ) = \frac{1}{\sqrt{L}}[1 ~~ e^{j 2\pi \frac{d}{\lambda } \sin \phi } ~~ \dots ~~ e^{j 2\pi \frac{d}{\lambda } (L-1) \sin \phi }]^T \end{aligned}$$

where $\phi$ is the AoA or AoD according to the channel’s type. In addition, $\Lambda$ denotes the wavelength, while *d* is the inter-element spacing between antennas.

#### Path loss model

The path loss for each link is modeled as:

12 $$\begin{aligned} \beta _{t,u}&= \left( \frac{\lambda }{4 \pi d_{t,u}}\right) ^n \end{aligned}$$

13 $$\begin{aligned} \beta _{t,r}&= \left( \frac{\lambda }{4 \pi d_{t,r}}\right) ^n \end{aligned}$$

14 $$\begin{aligned} \beta _{r,u}&= \left( \frac{\lambda }{4 \pi d_{r,u}}\right) ^n \end{aligned}$$

where $d_{t,u},d_{t,r}, \text {and}, d_{r,u}$ are the distances between the BS and user in the direct link, BS-RIS, and RIS-user, respectively. $\lambda$ is the carrier wavelength and *n* is the path loss exponent.

### Data detection

The received signal $y_k$ at the user *k* according to Eq. (1) can be rewritten by:

15 $$\begin{aligned} \begin{aligned}&y_k = \textbf{h}_{\text {eff},k}\textbf{x} + n_k = \underbrace{\textbf{h}_{\text {eff},k} \textbf{w}_k s_k}_{\text {Desired Signal}} + \\&\underbrace{\sum _{j \ne k}^K \textbf{h}_{\text {eff},k}\textbf{w}_j s_j}_{\text {Interference}} + n_k \end{aligned} \end{aligned}$$

The effective channel vector $\textbf{h}_{\text {eff},k} \in \mathbb {C}^{1 \times L_S}$ for the *k*-th user is defined as

16 $$\begin{aligned} \textbf{h}_{\text {eff},k} = \textbf{h}_k \textbf{AS}, \end{aligned}$$

where $\textbf{h}_k \in \mathbb {C}^{1 \times L_S}$ is the composite channel vector from the transmitter (including both the direct and IRS-assisted components) to the *k*-th user, and $\textbf{AS} = \textrm{diag}(\textbf{a})$ is the antenna selection matrix defined in subsection 2.1.

To exploit the NOMA principle, we assume the users are ordered based on their effective channel power gain, where the user $U_1$ has the weakest channel and $U_K$ has the strongest channel. Consequently, the successive interference cancellation (SIC) is applied to the receiver as follows:

- **Decoding** $U_k$: The SINR for a user *k* to extract its own signal $s_k$, after successfully canceling the signals of $U_1$ is given by:
  17 $$\begin{aligned} \gamma _k = \frac{|\textbf{h}_{\text {eff},k} \textbf{w}_k|^2}{\sum _{j=1}^{k-1} |\textbf{h}_{\text {eff},k} \textbf{w}_j|^2 + \sigma ^2} \end{aligned}$$
- **Decoding** $U_1$: Additionally, the user *k* must be able to decode the signals of all stronger users $j \in \{k+1, \ldots , K\}$ before canceling them. The SINR for user *k* to decode the signal $s_j$ (where $j > k$) is:
  18 $$\begin{aligned} \gamma _{k \rightarrow j} = \frac{|\textbf{h}_{\text {eff},k} \textbf{w}_j|^2}{\sum _{l=1}^{j-1} |\textbf{h}_{\text {eff},k}\textbf{w}_l|^2 + \sigma ^2} \end{aligned}$$

next The achievable rate for user *k* is given by

19 $$\begin{aligned} R_k = \log _2(1 + \gamma _{k}) \end{aligned}$$

## Joint optimization problem

The objective is to maximize the system’s Sum Rate SE by jointly optimizing the Power Allocation ($\textbf{W}$), Antenna Selection ($\textbf{AS}$), and RIS Phase Shifts (${\theta }$), subject to constraints. The joint optimization problem is solved using an AQSA framework combined with an alternating optimization strategy. The optimization problem can be expressed as:

20 $$\begin{aligned} \begin{aligned} \underset{\textbf{W}, \textbf{AS}, {\theta }}{\text {maximize}}&\quad J(\textbf{W}, \textbf{AS}, {\theta }) = \sum _{k=1}^{K} R_k \\ \text {subject to}&\quad \sum _{k=1}^{K} w_k \le P_{\text {max}} \\&\quad w_k \ge 0, \quad \forall k \in \{1, \dots , K\} \\&\quad AS_i \in \{0, 1\}, \quad \forall i \in \{1, \dots , L_{TX}\} \\&\quad \sum _{i=1}^{L_{TX}} AS_i = L_s \\&\quad |\boldsymbol{\Theta }_{j, j}| = 1, \quad \forall j \in \{1, \dots , L_s\} \\ \&\quad \theta _j \in [0, 2\pi ), \quad \forall j \in \{1, \dots , L_s\} \end{aligned} \end{aligned}$$

where the constraints are described according to Table 1

**Table 1:** Summary of Optimization Constraints.

Constraint	Explanation
$\sum _{k=1}^{K} w_k \le P_{\text {max}}$	The sum of the power allocated to all *K* users ($w_k$) must be less than or equal to the maximum total transmit power allowed at the Base Station ($P_{\text {max}}$).
$w_k \ge 0, \quad \forall k \in \{1, \dots , K\}$	The power allocated to each user ($w_k$) must be non-negative. This is a fundamental physical requirement.
$AS_i \in \{0, 1\}, \quad \forall i \in \{1, \dots , L_{TX}\}$	$AS_i$is a binary decision variable for transmit antenna *i*.$AS_i=1$means the antenna is active, and$AS_i=0$means it is inactive.
$\sum _{i=1}^{L_{TX}} AS_i = L_s$	The total number of active antennas must equal a specific, predefined number$L_s$. This limits complexity and power consumption.
$|\boldsymbol{\Theta }_{j, j}| = 1, \quad \forall j \in \{1, \dots , L_s\}$	The magnitude of the reflection coefficient for each RIS element ($\boldsymbol{\Theta }_{j, j}$) must be exactly one. This enforces that the RIS is passive and only changes the signal’s phase, without amplification.
$\theta _j \in [0, 2\pi ), \quad \forall j \in \{1, \dots , L_s\}$	The phase shift ($\theta _j$) applied by the *j*-th RIS element can be continuously adjusted over the full range, allowing for ideal beamforming control.

In addition, $R_k$ is the data rate for the *k*-th user, determined by the NOMA SIC procedure:

21 $$\begin{aligned} R_k = \log _2\left( 1 + \frac{G_k(\textbf{AS}, {\theta }) w_k}{G_k(\textbf{AS}, {\theta }) \sum _{j \in \mathcal {I}_k} w_j + \sigma ^2}\right) \end{aligned}$$

The effective channel power gain $G_k$ is calculated as the squared norm of the total effective channel vector $\textbf{h}_{\text {eff}, k}$:

$$\begin{aligned} G_k(\textbf{AS}, {\theta }) = \Vert \textbf{h}_{\text {eff}, k}\Vert ^2 \end{aligned}$$.

The joint optimization problem is introduced in Algorithm 1 where the initial solutions $\textbf{W}^{(0)}$, $\textbf{AS}^{(0)}$, and $\boldsymbol{\Theta }^{(0)}$ are chosen such that all problem constraints are satisfied:

- The precoding vector $\textbf{W}^{(0)}$ : $\sum _{k=1}^{K} w_k^{(0)} \le P_{\max }$ and $w_k^{(0)} \ge 0, \forall k$.
- The IRS phase shifts $\boldsymbol{\Theta }^{(0)}$ satisfy the unit-modulus constraints: $|\Theta _{j,j}^{(0)}| = 1$ and $\theta _j^{(0)} \in [0,2\pi ), \forall j$.
- The antenna selection vector $\textbf{AS}^{(0)}$ is binary and satisfies the selection constraint: $AS_i^{(0)} \in \{0,1\}$ and $\sum _{i=1}^{L_{TX}} AS_i^{(0)} = L_s$.

A simple feasible initialization can be obtained, for example, by allocating equal power to each user, randomly generating IRS phases on the unit circle, and randomly selecting $L_s$ active antennas.

**Algorithm 1:**
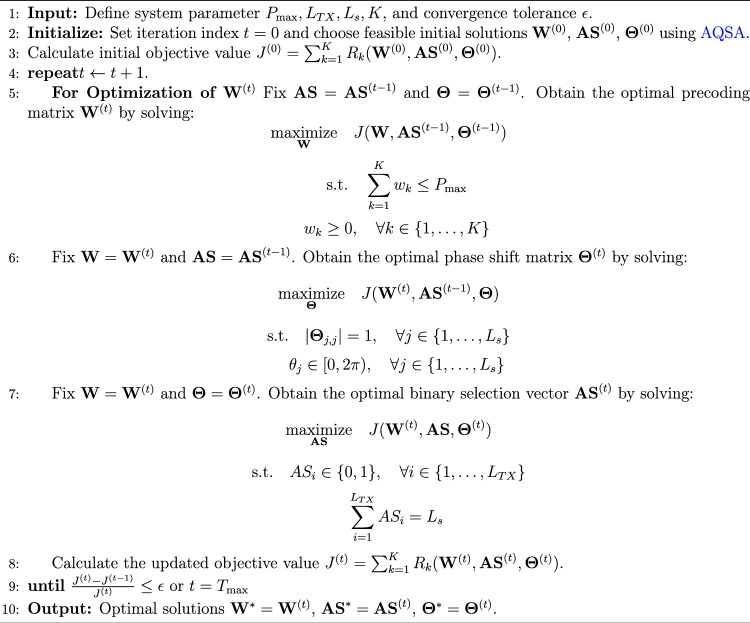
Joint Optimization Algorithm of AQSA

In Algorithm 1, AQSA is employed in each optimization step to handle the non-convex and discrete nature of the joint precoding, RIS phase shift, and antenna selection problem. Algorithm 1 adopts an alternating optimization framework, while the proposed AQSA algorithm is employed as the core solver for each subproblem as shown in Algorithm   2. Consequantly, within each optimization step of Algorithm 1, AQSA governs the generation, evaluation, and acceptance of candidate solutions.

### Convergence analysis

The suggested AQSA algorithm should find solutions that are close to the best ones because it can explore randomly and has an adaptive cooling mechanism. The way the proposed AQSA algorithm converges is based on the general idea of simulated annealing-based optimization. The algorithm lets worse solutions be accepted with a certain level of probability at higher temperatures. This helps avoid local optima and encourages global exploration. The adaptive cooling schedule slowly lowers the temperature, which makes it less likely that bad solutions will be accepted. This makes the search process focus on exploiting promising areas of the solution space. The optimization process terminates when one of the following stopping criteria is satisfied: (i) the maximum number of iterations is reached, or (ii) the improvement in the objective function becomes smaller than a predefined threshold over several consecutive iterations. The monotonic increase of the objective function ($J^{(t)} \ge J^{(t-1)}$) and the boundedness of the total system capacity ensure that the AQSA algorithm converges to at least a local optimum. The speed and quality of convergence depend heavily on the solution methods chosen for subproblems 2 and 3. Then, the EE of the system can be calculated after obtaining the sum rate as introduced in the next subsection.

### Energy efficiency

Energy efficiency is computed as the ratio between total achievable rate and total power consumption:

22 $$\begin{aligned} \text {EE} = \frac{\sum _{k=1}^{K} R_k}{P_{\text {tx}} + P_{\text {RIS}} + P_c+P_{RF}} \end{aligned}$$

where $P_{\text {tx}}$ is the total transmitted power, $P_{\text {RIS}}$ is the RIS power consumption, $P_c$ is the circuit power, and $P_{RF}$ is the radio frequency (RF) chain power consumption.

## Adaptive quantum-inspired simulated annealing

This section describes a hybrid version of the AQSA is designed to solve the joint problem of antenna selection, RIS phase shift assignment, and power allocation in a MIMO–NOMA system. This modified optimization algorithm is different from classical SA and quantum-inspired simulated annealing (QISA) algorithms. Unlike conventional QISA, which relies on a fixed cooling schedule, the proposed AQSA introduces an adaptive cooling mechanism that dynamically adjusts the temperature according to the improvement rate of the objective function. This adaptive strategy enables the algorithm to maintain exploration capability during early iterations while accelerating convergence in later stages. The quantum-inspired representation allows the algorithm to encode multiple candidate solutions simultaneously through probability amplitudes. This probabilistic representation improves the exploration ability of the search process and reduces the likelihood of premature convergence compared to classical binary encoding used in conventional SA-based algorithms. The optimization problem in RIS-assisted MIMO–NOMA systems is inherently high-dimensional due to the joint optimization of antenna selection, RIS phase shifts, and power allocation. Conventional optimization algorithms often suffer from premature convergence or excessive computational complexity when dealing with such large search spaces. The proposed AQSA algorithm addresses this challenge by combining quantum-inspired probabilistic representation with an adaptive cooling mechanism, enabling efficient exploration and faster convergence in large-scale optimization problems.

Table 2 summarizes the difference between SA, QISA, and AQSA. The aim of this work, as explained before, is to maximize a performance metric, SE or EE, while satisfying power and hardware constraints. For each transmit antenna $i\in \{1,\dots ,L_{\textrm{Tx}}\}$, a *Q-bit* is used as follows:

23 $$\begin{aligned} |\psi _i^{(a)}\rangle = \alpha _i^{(a)}|0\rangle + \beta _i^{(a)}|1\rangle ,~ |\alpha _i^{(a)}|^2 + |\beta _i^{(a)}|^2 = 1. \end{aligned}$$

where $\alpha _i \in \mathbb {C}$ is the probability amplitude of the Q-bit being in state 0 (no selection), while $\beta _i \in \mathbb {C}$ is the probability amplitude of the Q-bit being in state 1 (antenna selected). Measuring these Q-bits yields a binary antenna selection mask $x_i\in \{0,1\}$.

In addition, if the phases are discrete and chosen from a set $\Theta = \{0, \Delta \theta , 2\Delta \theta , \dots \}$, each RIS element can be represented by multiple Q-bits (binary encoding) or angle encoding. Moreover, If the phases are approximately continuous in $[0,2\pi )$, each element is represented by a real variable $\theta _j$, updated via Gaussian perturbation scaled by the temperature. In the same line, define the normalized power vector $\textbf{W}=[w_1,\dots ,w_K]$, where $p_k\in [0,1]$, and map it to actual transmit power via $w_k = w_k P_{\max }$ or under the constraint $\sum _k p_k \le P_{\max }$. The internal representation uses continuous variables updated by adaptive Gaussian perturbations based on temperature $T$. The steps of proposed optimization problems are as follows:

**Step 1**: Converting quantum states into solutions that can be evaluated. For antenna selection, measure each Q-bit according to the following equation:

24 $$\begin{aligned} a_i = {\left\{ \begin{array}{ll} 1 & \text {if } \text {rand} < |\beta _i^{(a)}|^2,\\ 0 & \text {otherwise.} \end{array}\right. } \end{aligned}$$

Consequently, RIS phases according to discrete representation, Measure the corresponding Q-bits to obtain a binary string, then map it to a phase value in $\Theta$.However, for continuous representation, take the real-valued vector $\boldsymbol{\theta }$ and apply Gaussian perturbation: $\theta _j' = \theta _j + \mathcal {N}(0,\sigma _T^2)$, where $\sigma _T$ scales with temperature $T$. Moreover, each continuous variable $w_k$ is measured directly and normalized to satisfy the total power constraint.

**Step 2**:Objective Function and Constraints The objective function (e.g., sum-rate) is evaluated under the generated solution in Eq. (20) The rate $R_k$ is computed using standard NOMA procedures (user ordering, SIC, etc.) with the given channel model.

**Step 3**:  Quantum Updates and Adaptive Operations

1. Q-bit Update for Antenna Selection: After measuring the solution and evaluating it, each Q-bit is updated using a quantum rotation gate:
  25 $$\begin{aligned} \begin{bmatrix}\alpha _i^{(a)\prime }\\ \beta _i^{(a)\prime }\end{bmatrix} = \begin{bmatrix} \cos (\Delta \theta _i^{(a)}) & -\sin (\Delta \theta _i^{(a)})\\ \sin (\Delta \theta _i^{(a)}) & \cos (\Delta \theta _i^{(a)}) \end{bmatrix} \begin{bmatrix}\alpha _i^{(a)}\\ \beta _i^{(a)}\end{bmatrix} \end{aligned}$$
  where the adaptive rotation angle is defined as:
  26 $$\begin{aligned} \Delta \theta _i^{(a)} = \Delta \theta _{\max }\exp \!\Big (-\frac{E_{\text {best}}-E_i}{T}\Big ) \cdot s_i \end{aligned}$$
  with $s_i\in \{+1,-1\}$ demonstrate the rotation direction depending on whether the bit in the best solution is active or not.
2. RIS Phase Updates: Apply the same Q-bit mechanism for each element, then map the bits to phase values.
  27 $$\begin{aligned} \theta _j' = \theta _j + \mathcal {N}\big (0,\sigma _T^2\big ),\qquad \sigma _T = \sigma _{\max }\frac{T}{T_{\text {init}}} \end{aligned}$$
  followed by phase normalization $\theta _j'\leftarrow \textrm{mod}(\theta _j',2\pi )$.
3. NOMA Power allocation Updates Update the power vector with continuous perturbation proportional to $T$ as the following:
  28 $$\begin{aligned} w_k' = w_k + \mathcal {N}\big (0,\nu _T^2\big ),\qquad \nu _T = \nu _{\max }\frac{T}{T_{\text {init}}} \end{aligned}$$
  Then normalize to satisfy the total power constraint:
  29 $$\begin{aligned} w_k^+ = \max (0, w_k'), \quad w_k = \frac{w_k^+}{\sum _j w_j^+} P_{\max }. \end{aligned}$$

**Step 4:** Acceptance Rules and Adaptive Cooling, after generating a new solution, compute the energy difference:

30 $$\begin{aligned} \Delta E = J_{\text {curr}} - J_{\text {new}}. \end{aligned}$$

The SA-based acceptance probability is:

31 $$\begin{aligned} P_{\text {accept}} = {\left\{ \begin{array}{ll} 1, & \Delta E \le 0\\ \exp \!\Big (-\dfrac{\Delta E}{T}\Big ), & \text {otherwise.} \end{array}\right. } \end{aligned}$$

Adaptive cooling is based on the acceptance rate $\text {AR}$:

32 $$\begin{aligned} \text {AR} = \dfrac{\#\text {accepted}}{M}. \end{aligned}$$

The cooling factor $\alpha$ is updated as the following:

33 $$\begin{aligned} \alpha \leftarrow {\left\{ \begin{array}{ll} \min (\alpha + \delta , \alpha _{\max }), & \text {if }\text {AR}>\text {AR}_{\text {high}}\\ \max (\alpha - \delta , \alpha _{\min }), & \text {if }\text {AR}<\text {AR}_{\text {low}}\\ \alpha , & \text {otherwise.} \end{array}\right. } \end{aligned}$$

Then $T \leftarrow \alpha T$. Light reheating can also be applied if $J_{\text {best}}$ does not improve over many iterations. The general steps of AQSA algorithm are presented in Algorithm 2

where the parameters can be defined as: $\textbf{S}$ Candidates antenna–RIS configuration (binary vector or selection mask), $\textbf{S}_{\text {init}}$ denotes the initial solution used to start the search process, while $\textbf{S}_{\text {curr}}$ is the current solution being evaluated. In addition, $\textbf{S}_{\text {new}}$ present the new solution generated after perturbation, which $\textbf{S}_{\text {best}}$ gives the best solution found during optimization, while $J_{\text {best}}$ is the maximum objective function value. However, $\text {Objective}(\cdot )$ present mapping from configuration $\textbf{S}$ to the performance metric. The *E* is the energy value used by SA ($E = -J$), and $T_{\text {initial}}$ is the initial (highest) temperature, while $T_{\text {final}}$ is the final (minimum) temperature, and *T* is the current temperature. additionally, $\alpha$ indicates the cooling factor controls the rate of temperature decay, $\alpha _{\text {initial}}$ is the initial cooling factor. $\alpha _{\min } \text {and} \alpha _{\max }$ are lower and upper limits on the cooling rate, respectively. $\delta$ is the step size used to adaptively update $\alpha$. *M* is the number of iterations performed at each temperature level. $\text {AR}$ gives the acceptance rate of new solutions in each temperature loop, while $\text {AR}_{\text {high}}$ and $\text {AR}_{\text {low}}$ are the thresholds for controlling adaptation of the cooling factor. $\text {rand}(0,1)$ is the random number used for acceptance testing. Finally, $\epsilon _T$ is a temperature-dependent perturbation scaling factor.

**Table 2:** Comparison of Simulated Annealing (SA), Quantum-Inspired SA (QISA), and Adaptive QISA (AQSA).

Aspect	Traditional SA	QISA	AQSA
**Cooling Schedule**	Geometric (Fixed Rate): $T_{\text {new}} = \alpha \cdot T_{\text {old}}$, $\alpha$ is fixed (e.g., 0.95).	Geometric (Fixed Rate): $T_{\text {new}} = \alpha \cdot T_{\text {old}}$.	Adaptive: $T_{\text {new}} = \alpha _{\text {curr}} \cdot T_{\text {old}}$, where $\alpha _{\text {curr}}$ is dynamically adjusted based on the Acceptance Rate.
**Acceptance Rule**	Boltzmann: $P_{\text {accept}} = e^{-\Delta E / T}$.	Quantum-Inspired: $P_{\text {accept}} = \frac{1}{1 + e^{\Delta E / T}}$.	Quantum-Inspired: $P_{\text {accept}} = \frac{1}{1 + e^{\Delta E / T}}$.
**Search Behavior**	offers an opportunity to break free from local optima based on the Energy Difference ($\Delta E$).	Wider exploration is made possible by the gentler acceptance curve, which increases the likelihood of accepting worse solutions early on.	combines a clever, adaptable cooling mechanism with the robust exploration capabilities of QISA.
**Advantages**	Simplicity and conceptual clarity.	Better initial exploration than SA, especially useful for mixed continuous/discrete problems.	In order to increase the likelihood of discovering the global optimum prior to convergence, adaptive cooling makes sure the algorithm spends more time in efficient exploration regions (high acceptance rate).
**Expected Performance**	Excellent, but prone to get trapped in local optima if $\alpha$ is too forceful and sensitive to initial *T*.	superior to SA, but it may still converge too quickly if the fixed $\alpha$ is poorly chosen.	Highest Performance: Expected to produce the best outcome by skillfully striking a balance between the phases of exploration and exploitation.

**Algorithm 2:**
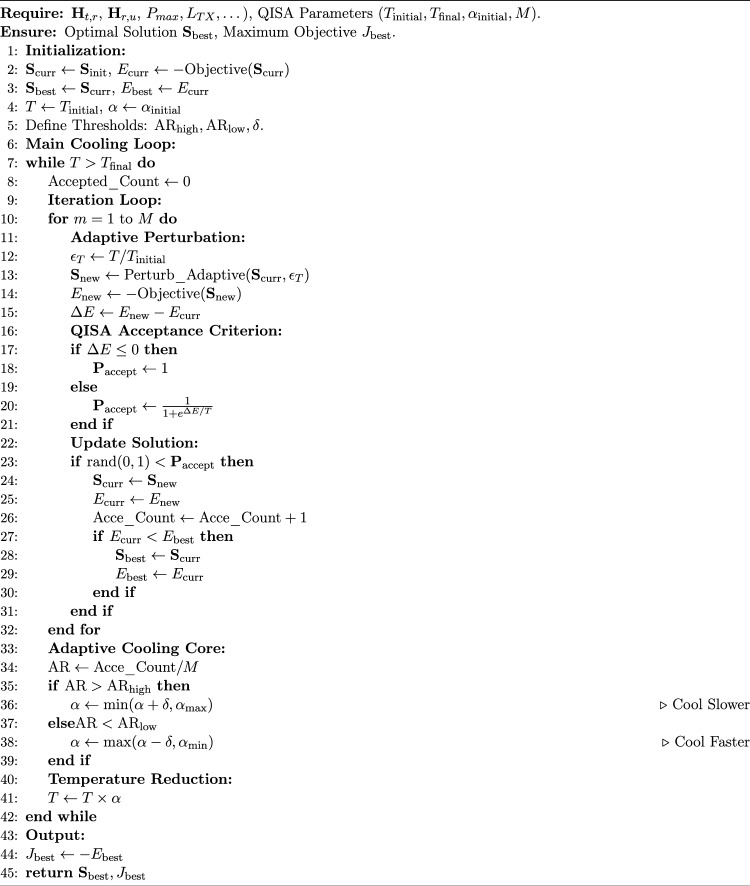
Adaptive Optimization of AQSA

## Simulation results

Extensive simulations were conducted to evaluate the performance of the proposed AQSA algorithm in RIS-assisted MIMO–NOMA systems operating at a carrier frequency of $f_c = 28~GHz$. The simulation results demonstrate that the proposed scheme achieves significant improvements in both spectral efficiency (SE) and energy efficiency (EE) compared with conventional optimization algorithms, including classical Simulated Annealing (SA), Particle Swarm Optimization (PSO), and Gray Wolf Optimization (GWO). Furthermore, the proposed AQSA-based joint optimization framework exhibits faster convergence and enhanced robustness under varying channel conditions and different user distributions. In addition, the integration of RIS effectively enhances the received signal strength and improves user fairness, highlighting its potential for improving system coverage and overall spectral efficiency. The proposed approach also maintains relatively low computational complexity, making it suitable for large-scale wireless systems and real-time implementation scenarios. The simulations are performed using $L_{Tx}=64$ transmit antennas (i.e., the total number of available antennas before the antenna selection process) and two users, each equipped with the same number of antennas. Table 3 summarizes the considered distances and corresponding path-loss values used in the proposed system model. The selected BS–RIS and RIS–UE distances are chosen to represent realistic deployment scenarios for the considered carrier frequency and the adopted path-loss model.

**Table 3:** Proposed distances and path loss values for RIS-assisted MIMO-NOMA system.

Link	*d* (m)	*n*	$\beta$
BS–User	50	3	$1.27\times 10^{-6}$
BS–RIS	30	2	$7.10\times 10^{-5}$
RIS–User	20	2.2	$2.30\times 10^{-4}$

**Figure 2:**
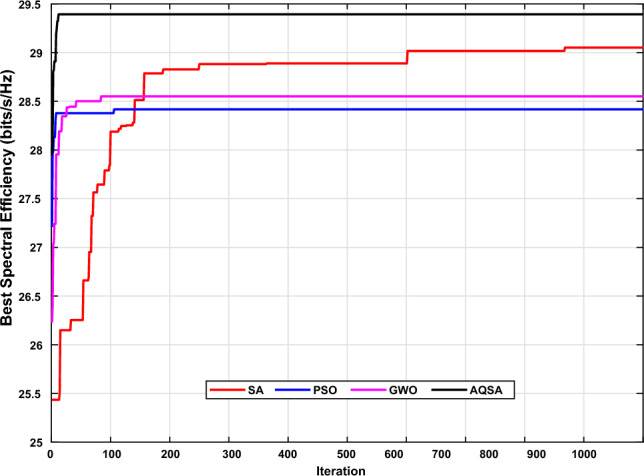
The convergence’s comparison between Proposed AQSA, PSO, SA, and GWO.

Fig. 2 shows the conversion speed (iterations) to reach the 29.62 SE. The proposed AQSA needs only 967 iterations, while each of PSO, SA, and GWO needs NaN iterations to reach the target SE. The best averages SE are as the following: AQSA=29.6313, SA=29.0514, PSO=28.4176, and GWO=28.5509 These results give an indication that the proposed scheme achieves the best result.

### Spectral efficiency evaluation

**Figure 3:**
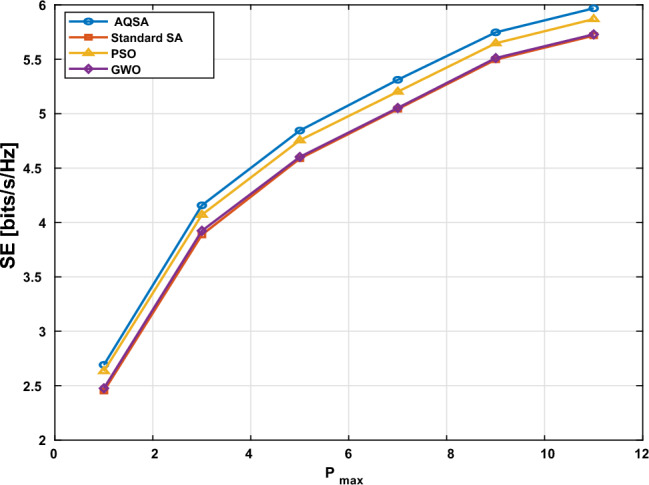
Spectral efficiency versus total transmit power $P_{max}$ with $L_{TX}=4,~ L_s=2,$and $L_{RIS}=16$ for $k=4$.

Following this approach, Fig. 3 illustrates the SE performance of different optimization algorithms under varying ($P_\textrm{max}$). It is clear that the proposed algorithm AQSA outperforms the other algorithms across all power levels. This better performance is due to AQSA’s adaptive cooling schedule and better perturbation strategy, which let it search the solution space more effectively and stay away from local optima. Consequently, as $P_\textrm{max}$ increases, the SE of all algorithms improves. due to the higher available transmit power. However, the gap between AQSA demonstrating the effectiveness of adaptive quantum-inspired simulated annealing in maximizing SE in RIS-assisted MIMO-NOMA systems.

**Figure 4:**
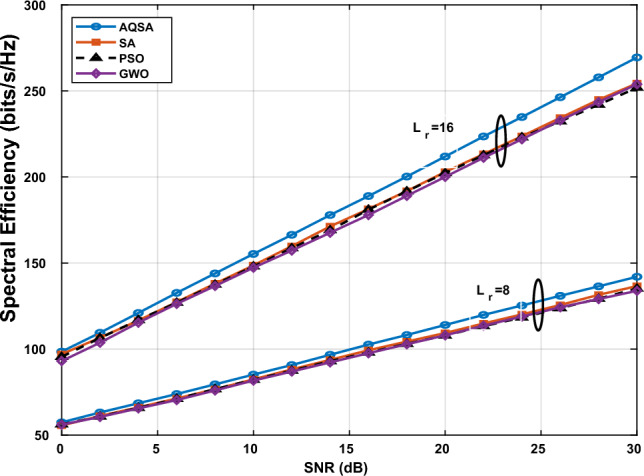
Spectral efficiency versus SNR with $L_{TX}=64,~ L_s=16,~\text {and}~ L_{RIS}=100$ for $L_r=8, 16$.

Additionally, Fig. 4 shows the spectral efficiency versus the SNR of the proposed optimization algorithm versus traditional optimizations SA, PSO, and GWO. The simulations are performed at a number of selected antennas—only $L_s=16$ antennas from 64 antennas. The number of RIS’s elements is $L_RIS=100$ and each user has number of received antennas equal to 4 and 8, so the total number of received antennas $L_r= 8,16$. As expected, SE increases monotonically with SNR for all algorithms and configurations. The configuration with $L_{r}=16$ consistently outperforms $L_{r}=8$, reflecting the gain from additional receive antennas in spatial multiplexing and diversity. Moreover, the proposed AQSA-based joint optimization achieves higher SE across the entire SNR range compared to benchmark methods (SA, PSO, GWO), with the largest advantage observed at moderate-to-high SNRs where interference management and phase optimization yield more pronounced benefits. These results confirm that (i) increasing $L_{r}$ and $L_{\textrm{RIS}}$ improves SE, and (ii) AQSA more effectively exploit the extra degrees of freedom provided by larger antenna/RIS configurations.

**Figure 5:**
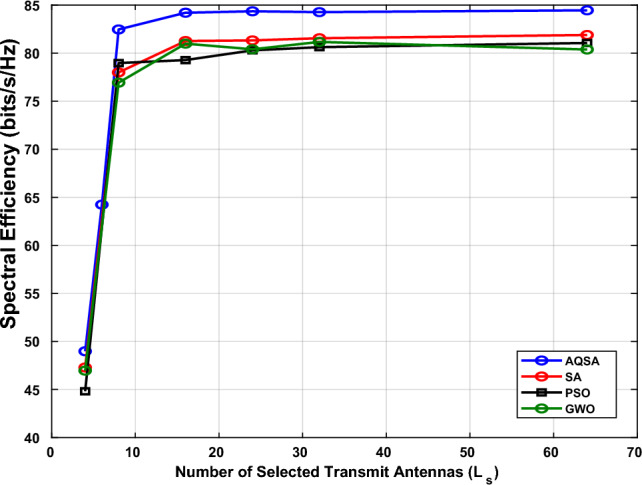
Spectral efficiency versus different numbers of selected antenna($L_s$) with $L_{TX}=64,~ L_{RIS}=100$,and $L_r=8$ at SNR=10 dB.

The fixed SNR reported in the following simulations refers to the transmit SNR, while the received SNR implicitly accounts for path loss and fading effects through the effective channel model. Consequently, Fig. 5 shows the SE versus different numbers of selected transmit antennas ($L_{s}$) for $L_{\textrm{Tx}}=64$, $L_{\textrm{RIS}}=100$, and $L_{r}=8$ at an SNR of 10 dB. SE increases with $L_{s}$ for all algorithms, reflecting the improved spatial multiplexing gain achieved by activating more antennas. Notably, the proposed AQSA-based joint optimization consistently outperforms the benchmark methods (SA, PSO, and GWO), demonstrating its ability to efficiently exploit the available antenna subset to maximize SE even with a limited number of receive antennas. These results confirm that careful selection of $L_{s}$ is crucial for achieving higher spectral efficiency in RIS-assisted MIMO systems. It is also noticed that the SE of AQSA achieves roughly constant SE for selected antennas greater than 15, while the remaining algorithms are constant after 25 antennas. All algorithms show limited SE improvement, as the system is constrained by the low number of spatial channels.

**Figure 6:**
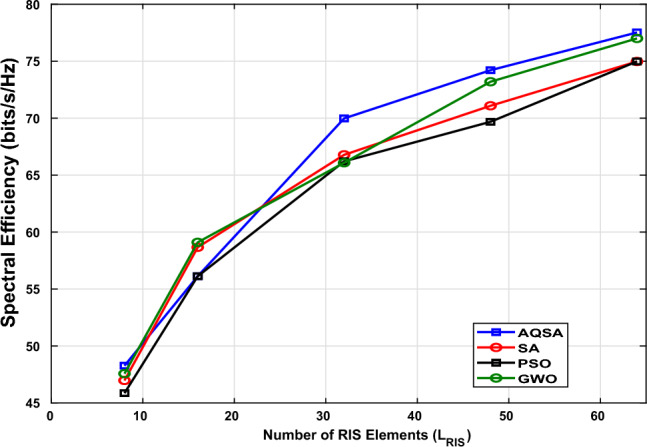
Spectral efficiency versus different numbers of RIS elements ($L_{RIS}=[8, 16, 32, 48, 64]$) at $L_{TX}=64,~ L_s=16$, and $L_r=8$ at SNR=10 dB.

Fig. 6 shows SE versus the different numbers of RIS elements. The simulations are performed at $L_s=16$ antennas with $L_r=8$ SNR=10 dB. The figure depicts that SE increases with the number of RIS elements for all algorithms, illustrating the role of RIS in providing additional passive beamforming gains and enhancing the effective channel. The proposed AQSA-based joint optimization consistently achieves higher SE compared to benchmark methods (SA, PSO, and GWO) after the number of RIS elements increases to more than 32 elements. These results confirm that increasing the number of RIS elements significantly improves performance SE and that AQSA can more effectively exploit the extra degrees of freedom provided by larger RIS configurations.

**Figure 7:**
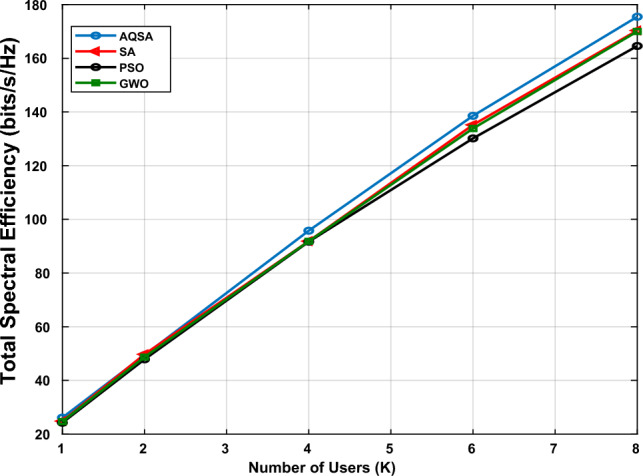
Spectral efficiency versus different numbers users($K=[1, 2, 4, 6, 8]$) at $L_{TX}=64,~ L_s=16,~L_{RIS}=32$,and $L_r/user=2$ at SNR=20 dB.

Consequently, Fig. 7 presents the comparison between AQSA and other algorithms in comparison (PSO, GWO, SA) at different numbers of users. The simulation parameters are used in simulations $L_{TX}=64$, $L_s=16$, and the number of recieve antennas for each user is  2 elements). These simulations are at SNR=20 dB. As expected, SE initially increases with the number of users due to the multiuser spatial multiplexing gain. However, the rate of improvement diminishes as *K* grows, reflecting the increased inter-user interference and the limited number of receiving antennas per user. Notably, the proposed AQSA-based joint optimization consistently outperforms benchmark methods (SA, PSO, and GWO) across all user numbers, demonstrating its effectiveness in managing interference and optimizing phase shifts in multiuser RIS-assisted MIMO systems. These results highlight the trade-off between user multiplexing and interference and confirm that AQSA can efficiently exploit system resources to maximize spectral efficiency even in dense user scenarios.

**Figure 8:**
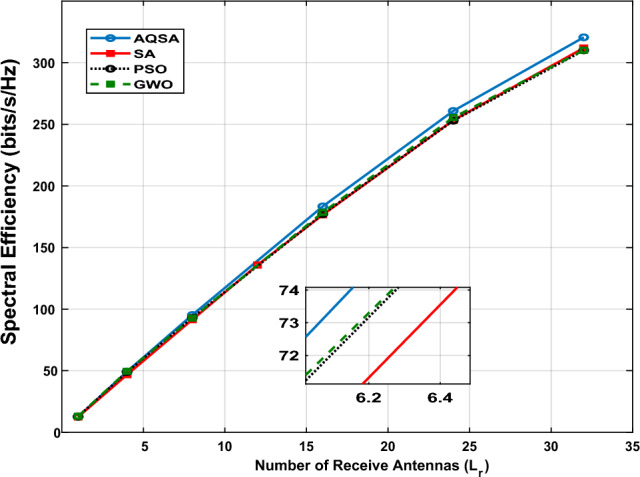
Spectral efficiency versus different numbers receive antennas($L_r=[1, 4, 8, 16, 24, 32]$) at $L_{TX}=64,~ L_s=32,~$ and $L_{RIS}=32$ at SNR=20 dB.

Fig. 8 presents SE as a function of the number of receiving antennas ($L_{r} = [1, 4, 8, 16, 24, 32]$) for $L_{\textrm{Tx}}=64$, $L_{s}=32$, and $L_{\textrm{RIS}}=32$ at an SNR of 20 dB. The results show a clear monotonic increase in SE with $L_{r}$ for all algorithms, which can be attributed to two key factors: (i) enhanced spatial multiplexing, allowing more parallel data streams to be reliably transmitted and received, and (ii) increased diversity gain, reducing the probability of deep fades in the wireless channel.

Notably, the performance gap between the proposed AQSA-based joint optimization and the benchmark methods (SA, PSO, and GWO) widens as $L_{r}$ increases. This indicates that AQSA is more capable of exploiting the additional degrees of freedom provided by larger receive arrays, effectively optimizing both antenna selection and RIS phase shifts. However, as $L_{r}$ reaches 16 and above, AQSA achieves significantly higher SE, demonstrating its robustness in leveraging large-scale MIMO benefits in RIS-assisted systems.

On the same line, Table 4 presents a detailed comparison of the achieved SE values for different optimization algorithms under various numbers of RIS elements. As observed, the proposed AQSA algorithm consistently outperforms the conventional SA, PSO, and GWO approaches. Specifically, AQSA achieves the highest SE of 77.52 bps/Hz when $L_{\textrm{RIS}}=64$, demonstrating its superior convergence behavior and exploration capability. Furthermore, all algorithms show a clear increase in SE as the number of RIS elements grows, confirming the positive impact of RIS-assisted reflection on system performance. These results verify that the proposed AQSA-based joint optimization effectively enhances spectral efficiency while maintaining computational efficiency.

The superior performance of the proposed AQSA algorithm can be attributed to its adaptive quantum-inspired search mechanism. Unlike conventional simulated annealing, which relies on a fixed cooling schedule and limited exploration capability, AQSA dynamically adjusts its temperature and mutation parameters according to the search progress. This adaptive behavior prevents premature convergence and enables a more balanced trade-off between exploration and exploitation. Moreover, the quantum-inspired representation enhances population diversity and allows the algorithm to escape local optima efficiently. Consequently, AQSA achieves faster convergence toward the global optimum solution, leading to higher spectral efficiency and more stable performance across different RIS configurations.

**Table 4:** Comparison of Spectral Efficiency (SE) for Different Algorithms at Different Number of $L_{RIS}$ at $L_{TX}=64, L_r=8, L_s=16,$and $SNR=10~dB$.

L_RIS_	PSO (SE)	GWO (SE)	AQSA (SE)	SA (SE)
8	46.9739	49.7922	48.2685	49.1979
16	58.6616	56.1626	56.1234	55.2526
32	66.7773	64.6096	69.9735	65.1836
48	71.0817	72.2433	74.2062	72.4379
64	74.9704	74.7918	77.5175	74.6063

Furthermore, Table 5 presents the spectral efficiency (SE) achieved by different algorithms—PSO, AQSA, SA, and GWO—for varying numbers of selected transmit antennas ($L_{s}$) with $L_{\textrm{Tx}}=64$, $L_{r}=8$, $L_{\textrm{RIS}}=32$, at an SNR of 10 dB. It can be observed that SE generally increases as $L_{s}$ grows, due to the improved spatial multiplexing gain provided by activating more antennas.

For small $L_{s}$ values (e.g., $L_{s}=4$), AQSA achieves slightly higher SE than the other algorithms, indicating its efficiency in selecting the most effective antennas under highly constrained configurations. As $L_{s}$ increases, all algorithms converge towards their maximum achievable SE, with AQSA consistently providing superior or comparable performance across all cases. Specifically, for moderate $L_{s}$ values (16–32), AQSA slightly outperforms PSO, SA, and GWO, reflecting its capability to jointly optimize antenna selection and RIS phase shifts to better exploit the system degrees of freedom. Interestingly, for very large $L_{s}$ (e.g., 64), the SE gains saturate, showing diminishing returns from activating additional antennas, which is expected due to the limited number of receive antennas ($L_{r}=8$). Overall, AQSA demonstrates robust performance, achieving the highest SE in most scenarios, highlighting its effectiveness in RIS-assisted MIMO systems even with a limited receiver array.

**Table 5:** Comparison of Spectral Efficiency (SE) for Different Algorithms at different number of $L_{s}$ at $L_{TX}=64, L_r=8, L_{RIS}=32,$and $SNR=10~dB$.

L_S_	PSO (SE)	AQSA (SE)	SA (SE)	GWO(SE)
4	47.2917	49.7922	45.5061	49.3007
8	77.9943	65.1641	75.0995	84.8642
16	81.2605	83.0374	80.3442	84.7749
24	81.3255	83.4755	79.219	83.1203
32	81.5508	83.8501	81.4017	83.6422
64	81.3884	84.1435	81.1035	82.2685

**Figure 9:**
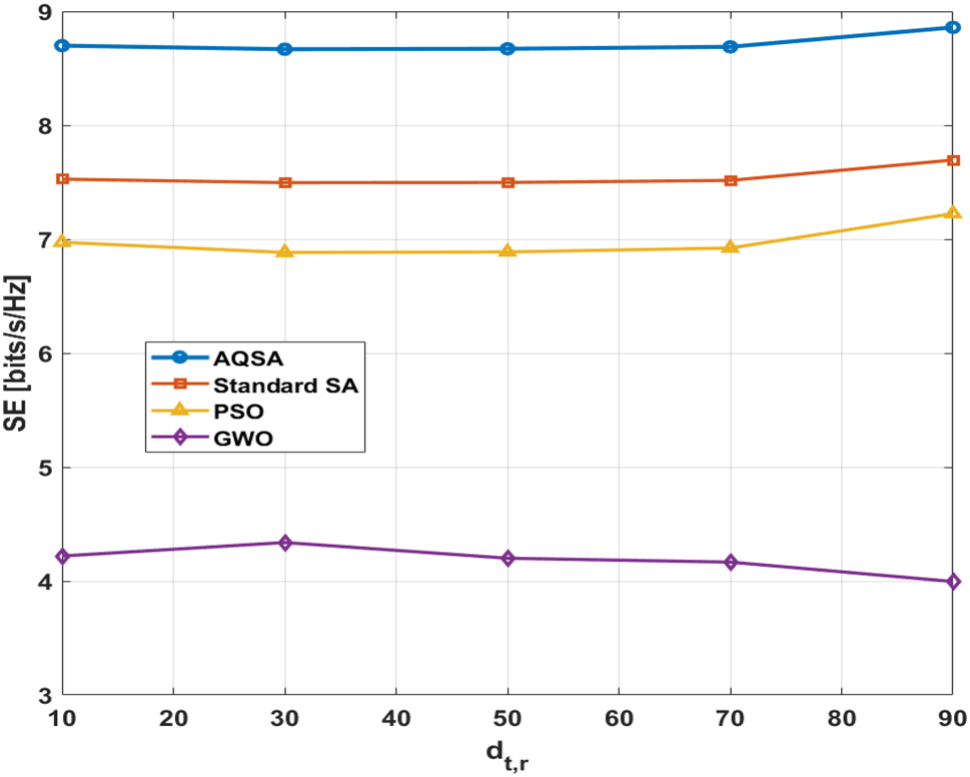
Spectral efficiency versus RIS Placement from BS at $L_{TX}=16,~ L_s=4,~\text {and}~ L_{RIS}=16, \sigma ^2=10^{-9}~dBm$ and  $P_{max}=10w$.

Moreover, Fig. 9 demonstrates that the proposed scheme is outperforms PSO, SA, and GWO. The simulation is to show SE against $d_{t,r}$ which denotes the distance between BS and RIS. While the distance between RIS and the user in simulation is given by $d_{r,u}= d_{t,u}-d_{t,r}$.

Now, the evaluation of EE will be introduced in the following subsection.

### Energy efficiency evaluation

In this section, we begin to evaluate the performance of the proposed algorithms versus benchmark algorithms(GWO, PSO, SA) in terms of EE. The parameters used in simulations are listed in Table 6.

**Table 6:** Parameters used for EE Calculation.

Parameter	Value
$P_{\textrm{Tx}}$	1 W
$P_{\textrm{RIS}}$	10-50 mW/element
$P_{\textrm{RF}}$	100-200 mW
$P_{\text {circuit}}$	1-2 W
*B*	20 MHz

**Figure 10:**
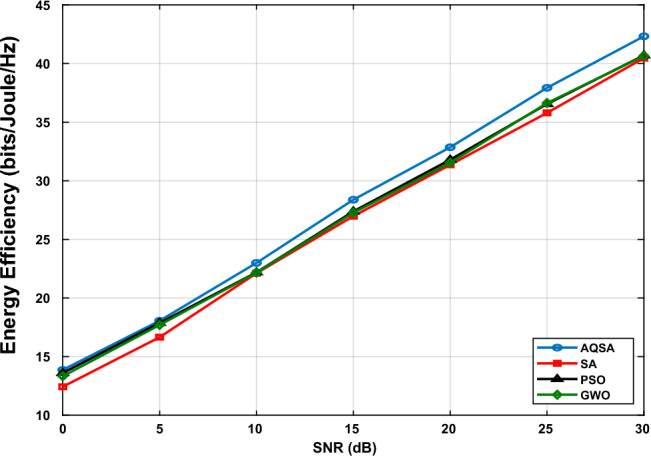
Energy efficiency versus SNR at $L_{TX}=64,~ L_s=16,~L_{RIS}=32$, and $L_r=8$.

Fig. 10 illustrates the EE as a function of SNR for $L_{\textrm{Tx}}=64$, $L_{s}=16$, $L_{\textrm{RIS}}=32$, and $L_{r}=8$. As observed, EE initially increases with SNR, reflecting the improved spectral efficiency and system throughput achieved at higher transmit power levels. However, the growth of EE slows down at high SNR values due to the increasing power consumption, which reduces the energy gain per transmitted bit. The proposed AQSA-based joint optimization consistently achieves higher EE compared to other methods (SA, PSO, and GWO) across the entire SNR range. This demonstrates that AQSA not only enhances SE but also effectively balances the trade-off between throughput and power consumption by optimally selecting antennas, allocating power to every user, and adjusting RIS phase shifts. The results emphasize that RIS-assisted MIMO systems benefit significantly from intelligent joint optimization strategies, which can maximize EE while maintaining high spectral performance.

**Figure 11:**
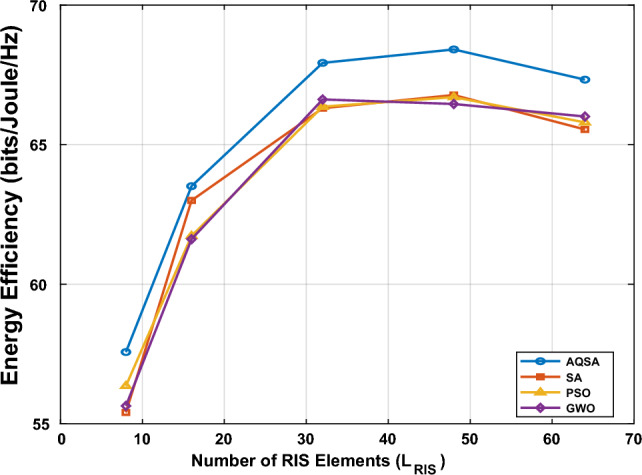
Energy efficiency versus $L_{RIS}=[8, 16, 32, 48, 64]$ at $L_{TX}=64,~ L_s=16,~L_r=8$, and SNR=15 dB.

Fig. 11 illustrates the EE performance of the proposed AQRA algorithm in comparison to the algorithms (BA, PSO, and GWO) against the number of RIS elements ($L_{RIS}$). A key observation is the concave relationship between $L_{RIS}$ and EE for all algorithms, which is a result of the trade-off between the array gain, which increases with $L_{RIS}$, and the associated power consumption of the RIS control circuitry. The EE for all schemes rises sharply up to $L_{RIS} = 32$ and reaches its maximum in the range of $L_{RIS} = 48$ to 64, confirming the existence of an optimal RIS size for maximizing EE. Crucially, the proposed AQRA consistently achieves the highest EE across the entire range of $L_{RIS}$ values, peaking at approximately 69.2 bits/Joule/Hz. This superior performance, particularly noticeable at lower $L_{RIS}$ values, validates the effectiveness of the AQRA approach in optimizing the passive beamforming for enhanced energy-efficient communication.

**Figure 12:**
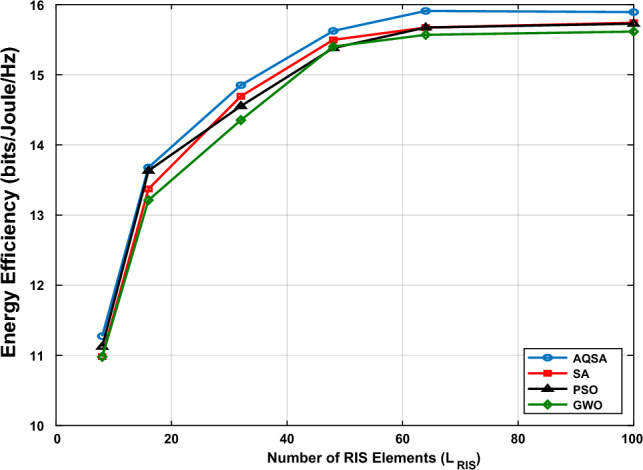
Energy efficiency versus $L_{RIS}=[8, 16, 32, 48, 64, 100]$ at $L_{TX}=64,~ L_s=32,~L_r=8$, and SNR=10 dB.

Consequently, Fig. 12 illustrates the EE performance of the proposed AQRA algorithm against the traditional algorithms (BA, PSO, and GWO) at the different number of RIS elements ($L_{RIS}$). The simulations are performed at $L_s=32$ elements, $L_r=8$ and $SNR=10~dB$. A key observation is the concave relationship between $L_{RIS}$ and EE for all algorithms, which is a result of the trade-off between the array gain, which increases with $L_{RIS}$, and the power consumption. The EE for all schemes rises sharply up to $L_{RIS} = 64$ and reaches its maximum value at $L_{RIS} =64$, and remains roughly constant. This result confirms the existence of balance between SE and power consumption, which makes the EE remains constant.Furthermore, for the whole range of $L_{RIS}$ values, the suggested AQRA consistently obtains the greatest EE. This improved performance, which is especially apparent at lower $L_{RIS}$ values, confirms that the AQRA method is successful in maximizing passive beamforming for more energy-efficient communication.

**Figure 13:**
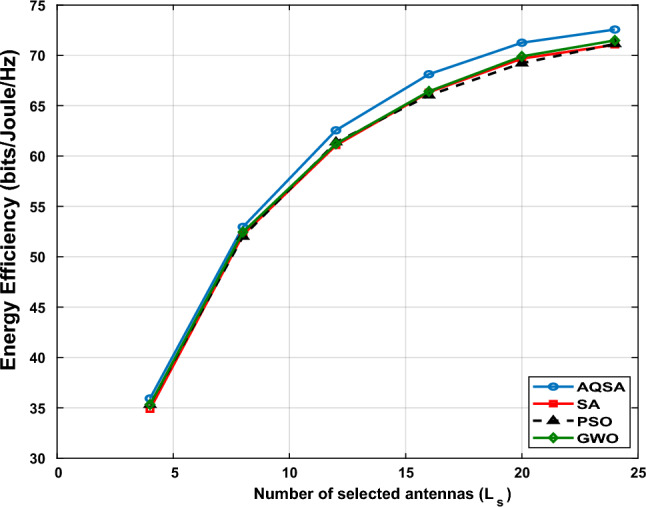
Energy efficiency versus $L_{s}=[4, 8, 12, 16, 20, 24, 32, 64]$ at $L_{TX}=64,~ L_{RIS}=32,~L_r=8$, and SNR=20 dB.

At an SNR of 20 dB, Fig. 13 shows the EE as a function of the number of chosen transmit antennas ($L_s = [4, 8, 12, 16, 20, 24, 32, 64]$) for $L_{\textrm{Tx}}=64$, $L_{\textrm{RIS}}=32$, and $L_r=8$. The findings demonstrate that EE first rises with $L_s$ as spectral efficiency and system throughput are improved by turning on additional antennas. However, EE starts to saturate or slightly decrease after a certain point (e.g., $L_s \ge 32$), as the throughput gain is outweighed by the additional power consumption from more active RF chains. The suggested AQSA-based joint optimization successfully selects the most advantageous antennas while balancing power consumption, consistently outperforming benchmark methods (SA, PSO, and GWO) across all $L_s$ values. This demonstrates how antenna selection and EE are traded off, with careful optimization able to maintain high EE without turning on superfluous transmit chains. The findings demonstrate that AQSA is especially successful in situations involving moderate to large numbers of antennas since it fully utilizes the RIS-assisted channel gains while optimizing the system’s energy efficiency.

Overall, the simulation results demonstrate that the proposed AQSA-based joint optimization framework effectively enhances SE, EE, and user fairness across various system configurations, highlighting its robustness, scalability, and practical applicability for large-scale RIS-assisted MIMO–NOMA systems.

### SIC decoding

**Figure 14:**
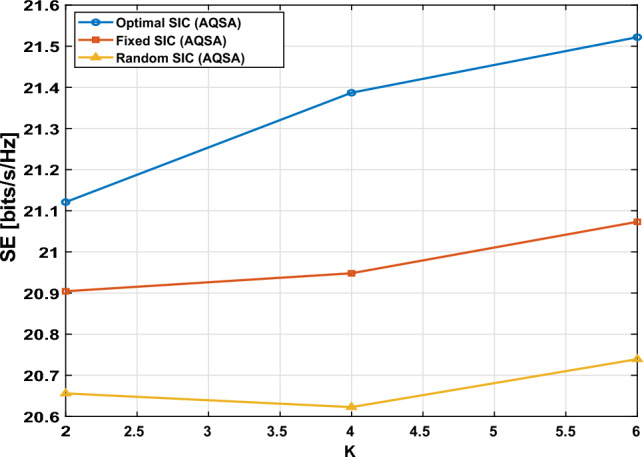
spectral efficiency versus different number of users for Proposed AQSA at $L_{TX}=4, L_{RIS}=16,$and $L_s=2$.

In NOMA, multiple users share the same frequency resource, distinguished in the power domain. At the receiver, each user applies the SIC strategy to reconstruct its signal. As shown in Fig. 14, using the AQSA algorithm, SE is a function of the number of users *k*under various NOMA SIC decoding orders. We compare three decoding strategies (random SIC, fixed SIC, and optimal SIC). Because the decoding order is chosen to maximize the sum-rate by carefully selecting which user is decoded at each step. From the result, it is clear that the optimal SIC curve consistently achieves the highest SE. The fixed SIC curve leads to a somewhat lower SE, particularly as the number of users increases. Finally, the Random SIC method demonstrates the lowest SE on average because the decoding order is not optimized, resulting in increased interference for some users. Table 7 presents the comparison between three different SIC scenarios. The SIC decoding orders are briefly explained as the following:

- *Optimal SIC:* Maximize the sum-rate by searching over all possible permutations of users:
  34 $$\begin{aligned} R_\textrm{sum}^*= \max _{\pi \in S_K} \sum _{i=1}^{K} R_{\pi (i)} \end{aligned}$$
  where $S_K$ is the set of all *K* permutations and $\pi (i)$ is the user index that is decoded at the *i*-th step.
- *Fixed SIC:* Predefined decoding order (e.g., $U_1 \rightarrow U_2 \rightarrow \dots \rightarrow U_K$):
  35 $$\begin{aligned} \pi _\textrm{fixed} = [1,2,\dots ,K] \end{aligned}$$
- *Random SIC:* Randomly choose a permutation of users in each Monte Carlo run:
  36 $$\begin{aligned} \pi _\textrm{random} \sim \text {Uniform Permutation}\{1,\dots ,K\} \end{aligned}$$

**Table 7:** Comparison of SIC decoding orders.

Type	Complexity	SE
Optimal	High (*O*(*K*))	Highest
Fixed	Low	Medium
Random	Low	Lowest

**Figure 15:**
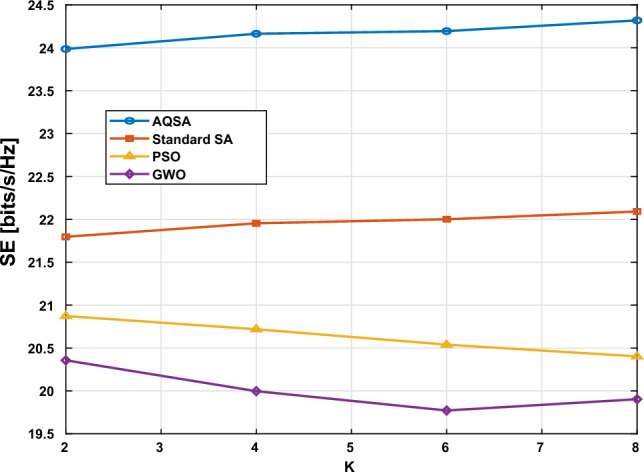
Spectral efficiency versus different numbers of users for optimal SIC at $L_{TX}=4, L_{RIS}=16, L_s=2, P_{max}=10W$ and $\sigma ^2=10^-3$.

In addition, Fig. 15 presents the comparison between different algorithms against the proposed ASQA. The simulations are done for optimal SIC to calculate SE at different numbers of users. The results demonstrate that the proposed scheme outperforms other algorithms in comparison. Table 8 shows the summarization of the results. The AQSA achieves at least an enhancement in SE by about 2 bps/Hz.

**Table 8:** SE Comparison for Different Algorithms.

K	AQSA	SA	PSO	GWO
2	23.986	21.797	20.873	20.357
4	24.164	21.954	20.719	19.997
6	24.195	22.001	20.539	19.772
8	24.320	22.092	20.402	19.903

### Complexity

Table 9 introduces the complexity comparison between proposed scheme and other algorithms like SA, PSO , and GWO. The proposed AQSA achieves highest SE against all tested algorithms while maintaining the lowest computational complexity. AQSA significantly reduces runtime compared to standard SA, PSO, and GWO. Notably, while PSO and GWO involve multiple particle/wolf evaluations per iteration, AQSA requires fewer iterations and fewer operations per iteration, providing an excellent trade-off between performance and efficiency. Given *P* and *W* are the swarm and wolf population sizes for PSO and GWO, repectively. $N_\textrm{perturb}$ is the Number of perturbations per iteration (SA/AQSA) ans $N_\mathrm{local\_tweak}$ denotes the Number of local tweaks per iteration (AQSA)

**Table 9:** Computational Complexity Comparison.

Algorithm	Complexity
AQSA	$O\Big ( N_\mathrm{iter_AQSA} \cdot (N_\textrm{perturb} + N_\mathrm{local\_tweak}) \cdot K \cdot L_{RIS}\cdot L_s \Big )$
SA	$O\Big ( N_\mathrm{iter_SA} \cdot N_\textrm{perturb} \cdot K \cdot L_{RIS} \cdot L_s \Big )$
PSO	$O\Big ( N_\mathrm{iter\_swarm} \cdot P_\textrm{swarm} \cdot K \cdot L_{RIS} \cdot L_s \Big )$
GWO	$O\Big ( N_\mathrm{iter\_swarm} \cdot W_\textrm{wolves} \cdot K \cdot L_{RIS} \cdot L-s \Big )$

## Conclusion

MIMO systems based on NOMA and RIS are described in this paper. Along with antenna selection, a simultaneous optimization of power allocation and RIS phase shift configuration is aimed at improving spectrum efficiency and system performance. The AQSA is used in the optimization to achieve the trade-off between global search capability and computational complexity. The system model incorporates digital beamforming to improve signal directivity and interference reduction, as well as RISs to reconfigure the wireless propagation environment. According to the simulation findings, compared to conventional methods, the suggested algorithm provides a significant improvement in throughput, energy consumption, and system robustness. The enhancement is at least 2 bps/Hz in SE. The viability of intelligent optimization algorithms supported by RIS in a future wireless communication system has been confirmed in this article.

## Data Availability

All MATLAB simulation codes and generated datasets used to support the findings of this study are available from the corresponding author upon reasonable request.
